# Medical Interventions and Women's Perceptions of Respectful Intrapartum Care: A National Survey‐Based Cohort Study

**DOI:** 10.1111/1471-0528.18329

**Published:** 2025-08-11

**Authors:** Karin Johnson, Charlotte Elvander, Kari Johansson, Sissel Saltvedt, Malin Edqvist

**Affiliations:** ^1^ Clinical Epidemiology Division, Department of Medicine Solna Karolinska Institutet Stockholm Sweden; ^2^ Department of Women's Health and Allied Health Professionals Karolinska University Hospital Stockholm Sweden; ^3^ Department of Women's and Children's Health, Department of Medicine Solna Karolinska Institutet Stockholm Sweden

**Keywords:** epidural, episiotomy, medical interventions, national survey, oxytocin, register based study, respectful care

## Abstract

**Objective:**

To investigate whether women's perceptions of respectful intrapartum care are influenced by the use of epidural, oxytocin, or episiotomy.

**Design:**

National survey‐based cohort study using data from the Swedish Pregnancy Survey 8 weeks postpartum (NPS‐8) merged with Swedish Pregnancy Register (SPR) data.

**Setting:**

Sweden, 2022–2023.

**Population:**

Primiparous women with a singleton pregnancy, cephalic presentation, spontaneous onset of labour, a live fetus at ≥ 37 weeks gestation, and a vaginal birth.

**Methods:**

Associations between respectful care and epidural analgesia, oxytocin, or episiotomy and their combinations were analysed using logistic regression to estimate adjusted odds ratios (aOR) with 95% confidence intervals (CI).

**Main Outcome Measures:**

Four NPS‐8 items assessing women's perceptions of being treated respectfully, receiving support, being informed, and being involved in decision‐making during childbirth.

**Results:**

Of 34 111 women, 20 363 (59.7%) responded, with the majority feeling respected during childbirth. Compared with women who had spontaneous vaginal births, those with instrumental births more frequently reported inadequate support (17.7% vs. 11.0%), insufficient information (25.1% vs. 16.2%), and lack of involvement in decision‐making (29.2% vs. 17.1%). Among women with spontaneous vaginal births, those subjected to an episiotomy were less likely to report being involved in decision‐making (77.8% vs. 83.8%; aOR 0.60, 95% CI 0.50–0.73). The combination of episiotomy and oxytocin was associated with the lowest adjusted odds of involvement in decision‐making (77.0% vs. 84.6%; aOR 0.54, 95% CI 0.43–0.69).

**Conclusion:**

Women with spontaneous vaginal births were less likely to report being involved in decision‐making when they underwent an episiotomy, or if oxytocin augmentation was followed by an episiotomy.

## 1 Introduction

Over the past decade, global awareness has grown regarding the challenges of ensuring women respectful maternity care [1, 2, 3]. Experiencing disrespectful care during childbirth can result in profound and lasting consequences, including feelings of disempowerment, emotional trauma, postpartum depression, and post‐traumatic stress disorder [4, 5, 6]. These negative experiences may influence future reproductive decisions, such as preferences regarding mode of birth [7].

Respectful care during childbirth has been defined as care that maintains dignity, privacy, and confidentiality, ensures freedom from harm, and enables informed choice and continuous support during childbirth [8]. While less research on respectful care has been conducted in high‐income countries [9], existing studies suggest that in these contexts, respectful care is characterised by responsiveness to women's needs, adequate support, appropriate pain relief, informed consent, and involvement in decision‐making [2, 3, 9]. Findings from qualitative studies [10, 11] indicate that not all Swedish women experience respectful care; however, the prevalence of disrespectful versus respectful care remains unknown.

Maternal risk factors for experiencing disrespectful care include lower educational attainment, certain ethnic groups, younger age, marital status, and excessive overweight [1, 12, 13, 14]. Additionally, medical interventions during childbirth, such as unplanned caesarean section (CS), induction of labour, episiotomy, epidural analgesia, and augmentation with oxytocin, have been shown to affect women's experiences of respectful care negatively [15, 16, 17].

The use of medical interventions during labour following spontaneous labour onset has increased substantially in Sweden over the past decades and is particularly common among primiparous women [18]. In 2023, 60% of first‐time mothers with spontaneous labour onset received epidural analgesia, while 54% had their labour augmented [18]. However, the prevalence of episiotomy among primiparous women in 2023 was low compared to other Scandinavian and European countries [19], with rates of 34% in instrumental and 6% in spontaneous vaginal births [18]. Interventions during childbirth are often interconnected [20]. For example, epidural use is frequently followed by oxytocin augmentation [21, 22]. Whether this interplay of one or more interventions affects the experience of respectful care remains unexplored.

Since 2020, a national survey, funded by the Swedish Association of Local Authorities and Regions (SALAR), has been distributed to women giving birth in Sweden. The survey examines care experiences, including whether women felt respected, received adequate support and information, and were involved in decision‐making during labour and birth [23].

With this study, we aimed to investigate whether women's perceptions of respectful intrapartum care are influenced by the use of certain medical interventions, including epidural, oxytocin, or episiotomy, either individually or in combination.

## 2 Methods

### 2.1 Study Design

This study uses data from the National Swedish Pregnancy Survey administered to women 8 weeks postpartum (NPS‐8) and the Swedish Pregnancy Register (SPR), a national quality registry covering 98.4% of all births in Sweden [24]. The NPS surveys are part of Sweden's National Maternity Care Initiative funded by SALAR [25], and comprise three surveys administered electronically to all pregnant women with a Swedish personal identification number at pregnancy week 25, at 8 weeks, and 1 year after birth. The questions cover patient‐reported experiences and outcome measures. Patient‐reported experiences include antenatal, labour, and postnatal care. Outcome measures include physical and mental health 1 year after birth. The NPS‐8 consists of 44 items assessing the experience of care during labour, birth and the immediate postpartum period (Data S1). The survey begins with a single item regarding perceived general health during the final trimester of pregnancy, followed by items related to antenatal care. The items in the survey were developed in an iterative process with input from women and caregivers incorporating their perspectives on the relevance and comprehensibility of the questions [26]. However, they have not been validated in terms of their psychometric properties. The NPS surveys are available in Swedish and in the most common languages among foreign‐born and immigrants in Sweden: English, Spanish, French, Finnish, Farsi, Arabic, and Somali. To increase participation, one reminder is sent after a week if the survey remains incomplete, and it remains open for a total of 6 weeks. Data collection for the surveys started on December 1st, 2020, and is ongoing. In our analytic sample, both completed surveys and partially completed surveys were included. Partially completed surveys were included if the woman had responded to at least the first four items of the NPS‐8.

### 2.2 Participants

The study population comprised primiparous women giving birth between 1 January 2022 and 31 December 2023 with term gestation (≥ 37 + 0 weeks), cephalic presentation, spontaneous start of labour, and a live vaginal birth who responded to the NPS‐8. Each survey response in the sample was then linked via the personal identification number to the individual women's data from SPR, including sociodemographic variables, pregnancy, and birth variables. Duplicate responses were removed. Women were not involved in the design, interpretation, or reporting of the findings in the present study.

### 2.3 Outcome Variables

To investigate the association between the use of the three chosen interventions under study and respectful care, four questions from the NPS‐8 were selected as outcome variables, which together represent respectful care: (1) “During labour and birth, did the caregivers treat you with respect?”; (2) “During labour and birth, did you receive support from the caregivers to the extent you desired?”; (3) “During labour and birth, did you receive enough information?”; (4) “During labour and birth, were you involved in planning and decision‐making to the extent you desired?”. These questions were selected as they are integral to the concept of respectful maternity care. All items are rated on a 5‐point Likert scale, ranging from 1; not at all to 5; completely. For all items, the response option “cannot/do not wish to answer” is provided, while the item regarding involvement in decision‐making additionally includes the response option “not applicable”. The four items representing the outcome variables were further dichotomised into a positive statement; Agree (response options 4–5) and a negative statement; Disagree (response options 1–3). Response options “cannot/do not wish to respond” or “not applicable”, were categorised as missing data.

### 2.4 Explanatory Variables

Epidural analgesia, augmentation with oxytocin, and episiotomy were chosen as explanatory variables and retrieved from the SPR. Oxytocin augmentation was identified using procedure codes DT036 and DT037 and the variable “Oxytocin during labour” (yes/no). Epidural analgesia was identified using the procedure code SN999, and episiotomy was identified using the variable “Episiotomy” (yes/no). Procedure codes refer to codes that record the type of medical treatment or procedure provided.

### 2.5 Covariates

Covariates were selected a priori to control for potential confounders. Information on all covariates was retrieved from the SPR. The following variables were considered as confounders and categorised accordingly: maternal age at birth (< 25 years, 25–29 years, 30–34 years, ≥ 35 years), early pregnancy body mass index (BMI; underweight < 18.5 kg/m^2^, normal weight 18.5–24.9 kg/m^2^, overweight 25–29.9 kg/m^2^, and obese ≥ 30 kg/m^2^), country of birth (Nordic/non‐Nordic), level of education (elementary school; none or < 9 years, upper secondary school; 12 years, university; > 12 years), pre‐pregnancy comorbidity (yes/no; hypertension, diabetes, asthma, systemic lupus erythematosus (SLE), endocrine disease, kidney disease, epilepsy), pregnancy comorbidity (yes/no; preeclampsia, pregnancy‐induced diabetes, hepathosis), mental illness (yes/no), positive self‐assessed health before pregnancy (yes/no), fear of birth (yes/no), gestational age at birth (continuous), and hospital size (< 1000 births/year, 1000–1999 births/year, 2000–2999 births/year, ≥ 3000 births/year, university hospital).

### 2.6 Statistical Analysis

Descriptive statistics, including means and standard deviations (SD) or numbers and percentages, were used to present maternal, labour, and birth characteristics as well as perceptions of respectful care of the women responding to the NPS‐8. To account for the impact of mode of birth on the four outcome variables reflecting respectful care [27, 28], we stratified the cohort accordingly. A Variance Inflation Factor test was performed to assess multicollinearity among covariates. BMI was the only covariate exceeding the threshold value of 5 [29], which was addressed using the continuous variable.

The associations between the outcome variables and epidural analgesia, oxytocin, or episiotomy and the combinations of (1) epidural and oxytocin, (2) epidural and episiotomy, (3) oxytocin and episiotomy, and (4) epidural, oxytocin, and episiotomy were examined using logistic regression to estimate odds ratios (OR) with 95% confidence intervals (CI). For all analyses, the significance level was set at < 0.05.

Given the established correlation between epidural analgesia and oxytocin augmentation, and the absence of such a relationship with episiotomy, two separate models were developed. Model A examined the association between epidural analgesia, oxytocin augmentation, and the four outcome variables related to respectful care. An interaction term was included in this model (epidural analgesia#oxytocin augmentation). Model B examined the association between the outcome variables and episiotomy individually, and in combination with (1) epidural analgesia, (2) oxytocin, or (3) epidural analgesia and oxytocin, and did not include an interaction term. Both models were adjusted for the following covariates: maternal age, BMI, country of birth, level of education, positive self‐assessed health before pregnancy, fear of birth, mental illness, pre‐pregnancy comorbidity, pregnancy comorbidity, gestational age at birth, and hospital size. Stata version 17.0 was used for all statistical analyses.

## 3 Results

The study cohort comprised 34 314 nulliparous women with spontaneous onset of labour and a vaginal birth between 2022 and 2023. Of these, 20 566 responded to the NPS‐8. After excluding 203 duplicate responses, the cohort included 34 111 women and 20 363 responses to the NPS‐8, giving a response rate of 59.7%. Since the survey data from the NPS‐8 included both fully and partially completed surveys, we chose to include all responses in which women had answered at least the first four items, resulting in a final study cohort of 18 994 women (Figure S1).

Women who responded to the NPS‐8 were significantly older (mean 30.1; SD 4.2 vs. mean 29.2; SD 4.6; *p* < 0.001), more often of Nordic origin (77.4% vs. 59.2%; *p* < 0.001) and more likely to have a higher educational level compared to non‐responders. However, there were no significant differences between responders and non‐responders regarding the use of epidural analgesia, oxytocin augmentation, or episiotomy (Table S1).

The majority of respondents (98.6%) completed the NPS‐8 in Swedish, and 87.8% had a spontaneous vaginal birth (Table 1). Furthermore, most women were aged 30–34 years, had a normal BMI, and identified as Nordic (Table 1). The stratification by mode of birth revealed that women who had a spontaneous vaginal birth were more likely to be younger (< 29 years) and obese, compared to those who had an instrumental birth (Table 1). Epidural analgesia was the most commonly used intervention, followed by oxytocin augmentation and episiotomy. The highest prevalence of interventions was observed among women who had an instrumental birth, with 73.5% using epidural analgesia for pain relief, 92.3% having their labours augmented with oxytocin, and 12.5% undergoing an episiotomy (Table 1). The vast majority of women reported being treated with respect, according to the first outcome variable in the NPS‐8, regardless of mode of birth. More pronounced differences were observed across the other outcome variables. Among women giving birth spontaneously, 10.1% reported feeling inadequately supported, compared to 17.7% of those who had an instrumental birth. Similarly, 14.9% of women in the spontaneous birth group indicated they had not received sufficient information, versus 25.1% in the instrumental birth group, whereas the corresponding rates for reporting lack of involvement in decision‐making were 15.4% and 29.2% respectively (Table 2). All four outcome variables reflecting respectful care had missing data between 4.6% and 5.2%. Those who did not answer were younger, had lower educational attainment, and were more often of non‐Nordic origin (Tables S1 and S2). The proportion of missing data for the four outcome variables was attributable to the inclusion of partially completed surveys. When restricting the analysis to fully completed surveys, the proportion of missing data was reduced to between 0.04% and 0.08%.

**TABLE 1:** Maternal and labour characteristics for 18 994 primiparous women with spontaneous start of labour and a spontaneous vaginal or instrumental birth responding to the NPS‐8, 2022–2023, Sweden.

Data from the SPR	Total population	Spontaneous vaginal birth	Instrumental birth	*p*
	*n* = 18 994	*n* = 16 677	*n* = 2317	
	*n* (%)	*n* (%)	*n* (%)	
Age (years), mean (SD)	30.2 (4.2)	30.1 (4.2)	31.1 (4.2)	
Age categories				
< 25	2044 (10.8)	1882 (11.3)	162 (7.0)	< 0.001
25–29	7054 (37.1)	6292 (37.7)	762 (32.9)	
30–34	7580 (39.9)	6580 (39.5)	1000 (43.2)	
≥ 35	2316 (12.2)	1923 (11.5)	393 (17.0)	
BMI (kg/m^2^), mean (SD)	24.6 (4.5)	24.7 (4.5)	24.3 (4.2)	
BMI categories				
Underweight (< 18.5)	422 (2.2)	370 (2.2)	52 (2.2)	0.008
Normal (18.5–24.9)	11 097 (58.4)	9691 (58.1)	1406 (60.7)	
Overweight (25–29.9)	4589 (24.2)	4033 (24.2)	556 (24.0)	
Obese (≥ 30)	2081 (11.0)	1873 (11.2)	208 (9.0)	
Missing data	805 (4.2)	710 (4.3)	95 (4.1)	
Level of education				
Elementary school	337 (1.8)	309 (1.9)	28 (1.2)	0.002
Upper secondary school	4712 (24.8)	4185 (25.1)	527 (22.7)	
University^a^	11 474 (60.4)	10 013 (60.0)	1461 (63.1)	
Missing data	2471 (13.0)	2170 (13.0)	301 (13.0)	
Living with partner				
Yes	17 187 (90.5)	15 077 (90.4)	2110 (91.1)	0.819
Missing data	604 (3.2)	542 (3.3)	62 (2.7)	
Country of birth				
Nordic	14 968 (78.8)	13 168 (79.0)	1800 (77.7)	0.439
Non‐Nordic	2514 (13.2)	2198 (13.2)	316 (13.6)	
Missing data	1512 (8.0)	1311 (7.9)	201 (8.7)	
Positive self‐assessed health before pregnancy				
Yes	14 935 (78.6)	13 095 (78.5)	1840 (79.4)	0.340
Missing data	2847 (15.0)	2508 (15.0)	339 (14.6)	
Pre‐pregnancy comorbidity^b^				
Yes	3825 (20.1)	3346 (20.1)	479 (20.7)	0.493
Pregnancy comorbidity^c^				
Yes	709 (3.7)	619 (3.7)	90 (3.9)	0.681
Mental illness				
Yes	4909 (25.9)	4325 (25.9)	584 (25.2)	0.423
Missing data	325 (1.7)	298 (1.7)	36 (1.6)	
Fear of birth				
Yes	1340 (7.1)	1164 (7.0)	176 (7.6)	0.263
Missing data	2213 (11.7)	1939 (11.6)	274 (11.8)	
Epidural analgesia				
Yes	10 985 (57.8)	9282 (55.7)	1703 (73.5)	< 0.001
Oxytocin augmentation				
Yes	10 871 (57.2)	8732 (52.4)	2139 (92.3)	< 0.001
Episiotomy				
Yes	1197 (6.3)	908 (5.4)	289 (12.5)	< 0.001
Missing data	613 (3.2)	548 (3.3)	65 (2.8)	
Hospital size^d^				
< 1000	1249 (6.6)	1088 (6.5)	161 (7.0)	0.472
1000–1999	4407 (23.2)	3848 (23.1)	559 (24.1)	
2000–2999	3519 (18.5)	3108 (18.6)	411 (17.7)	
≥ 3000	3672 (19.3)	3244 (19.5)	428 (18.5)	
University hospital	6143 (32.4)	5385 (32.3)	758 (32.7)	

^a^ 3–5 years.

^b^ Hypertension, diabetes type 1 or 2, systemic lupus erythematosus (SLE), renal conditions, and epilepsy.

^c^ Gestational diabetes, preeclampsia, and hepatopathy.

^**d**^ Annual birth volume and teaching status (44 labour wards included).

**TABLE 2:** Women's perceptions of respect, support, information, and involvement in decision‐making for 18 994 primiparous women with spontaneous start of labour and a spontaneous vaginal or instrumental birth, Sweden, 2022–2023.

Outcome variables from NPS‐8	Total population	Spontaneous vaginal birth	Instrumental birth	*p*
	*N* = 18 994	*n* = 16 677	*n* = 2317	
	*n* (%)	*n* (%)	*n* (%)	
During labour and birth, did the caregivers treat you with respect?^a^				
The caregivers treated me with respect	16 888 (88.9)	14 860 (89.1)	2028 (87.6)	< 0.001
The caregivers did not treat me with respect	1112 (5.9)	931 (5.6)	181 (7.8)	
Did not want to answer the question	45 (0.2)	40 (0.2)	5 (0.2)	
Missing data	949 (5.0)	846 (5.1)	103 (4.4)	
During labour and birth, did you receive support from the caregivers to the extent you desired?^a^				
I received support to the extent I desired	15 965 (84.1)	14 161 (84.9)	1804 (77.9)	< 0.001
I did not receive support to the extent I desired	2089 (11.0)	1678 (10.1)	411 (17.7)	
Did not want to answer the question	42 (0.2)	39 (0.2)	3 (0.1)	
Missing data	898 (4.7)	799 (4.8)	99 (4.3)	
During labour and birth, did you receive enough information?^a^				
I received enough information	14 770 (77.7)	13 159 (79.0)	1611 (69.5)	< 0.001
I did not receive enough information	3071 (16.2)	2490 (14.9)	581 (25.1)	
Did not want to answer the question	171 (0.9)	155 (0.9)	16 (0.7)	
Missing data	982 (5.2)	873 (5.2)	109 (4.7)	
During labour and birth, were you involved in planning and decision‐making to the extent you desired?^a^				
I was involved in planning and decisions to the desired extent	14 426 (76.0)	12 935 (77.6)	1491 (64.3)	< 0.001
I was not involved in planning and decisions to the desired extent	3246 (17.1)	2570 (15.4)	676 (29.2)	
Did not want to answer the question	132 (0.7)	110 (0.7)	21 (0.9)	
Not applicable	324 (1.7)	290 (1.7)	34 (1.5)	
Missing data	866 (4.6)	771 (4.6)	95 (4.1)	

^a^ All outcome variables were rated on a 5‐point Likert scale and dichotomised accordingly: agree (response options 4–5); disagree (response options 1–3).

### 3.1 Association Between Single Interventions and Respectful Care

Among women who had a spontaneous vaginal birth, those who received an epidural had higher odds of reporting involvement in decision‐making compared to those who did not (84.8% vs. 81.7%; aOR 1.33, 95% CI 1.21–1.47). In contrast, augmentation with oxytocin was associated with a 13%–18% reduction in the adjusted odds of women reporting that they received adequate support, sufficient information, or were involved in decision‐making compared to those who did not receive oxytocin. Notably, the absolute differences were small, especially the proportions related to oxytocin (1.7%–1.8%), suggesting a trend rather than clinically relevant differences (Table 3). Additionally, women who were subjected to an episiotomy, compared to those who were not, had decreased odds of reporting experiences of respectful care, particularly regarding sufficient information (79.7% vs. 84.5%; aOR 0.73, 95% CI 0.59–0.89) and involvement in decision‐making (77.8% vs. 83.8%; aOR 0.60, 95% CI 0.50–0.73) (Table 3).

**TABLE 3:** Crude and adjusted odds ratios (OR) with 95% confidence intervals (CI) for women's perceptions of respect, support, information, and involvement in decision‐making by use of medical interventions. Primiparous women with spontaneous labour onset and a spontaneous vaginal or instrumental birth, Sweden, 2022–2023.

	Spontaneous vaginal birth			Instrumental birth		
	*n* (%)	OR (95% CI)	aOR (95% CI)	n (%)	OR (95% CI)	aOR (95% CI)
Treated respectfully						
No epidural	6592/7041 (93.6)	1.0	1.0	543/587 (92.5)	1.0	1.0
Epidural	8268/8750 (94.5)	1.17 (1.02–1.33)	1.32 (1.13–1.54)	1485/1622 (91.6)	0.88 (0.62–1.25)	0.99 (0.66–1.50)
No oxytocin	7038/7509 (93.7)	1.0	1.0	158/172 (91.9)	1.0	1.0
Oxytocin	7822/8282 (94.5)	1.14 (1.00–1.30)	1.09 (0.93–1.27)	1870/2037 (91.8)	0.99 (0.56–1.75)	0.55 (0.24–1.30)
No episiotomy	13 562/14359 (94.2)	1.0	1.0	1713/1867 (91.8)	1.0	1.0
Episiotomy	811/866 (93.7)	0.91 (0.68–1.20)	0.84 (0.61–1.16)	259/278 (93.2)	1.23 (0.75–2.01)	1.56 (0.83–2.93)
Received support						
No epidural	6289/7059 (89.1)	1.0	1.0	471/589 (80.0)	1.0	1.0
Epidural	7872/8780 (89.7)	1.06 (0.96–1.17)	1.10 (0.98–1.24)	1333/1626 (82.0)	1.14 (0.90–1.45)	1.28 (0.98–1.69)
No oxytocin	6807/7535 (90.3)	1.0	1.0	148/174 (85.1)	1.0	1.0
Oxytocin	7354/8304 (88.6)	0.83 (0.75–0.92)	0.82 (0.73–0.93)	1656/2041 (81.1)	0.76 (0.49–1.16)	0.54 (0.30–0.96)
No episiotomy	12 930/14442 (89.5)	1.0	1.0	1518/1873 (81.1)	1.0	1.0
Episiotomy	762/869 (87.7)	0.83 (0.68–1.03)	0.74 (0.58–0.93)	234/278 (84.2)	1.24 (0.88–1.75)	1.27 (0.84–1.91)
Adequately informed						
No epidural	5769/6956 (83.3)	1.0	1.0	429/579 (74.1)	1.0	1.0
Epidural	7363/8693 (84.7)	1.11 (1.02–1.21)	1.10 (0.99–1.21)	1182/1613 (73.3)	0.96 (0.77–1.19)	1.10 (0.86–1.41)
No oxytocin	6310/7425 (85.0)	1.0	1.0	121/169 (71.6)	1.0	1.0
Oxytocin	6849/8224 (83.3)	0.88 (0.81–0.96)	0.84 (0.76–0.92)	1490/2023 (73.7)	1.11 (0.78–1.57)	1.02 (0.67–1.56)
No episiotomy	12 050/14263 (84.5)	1.0	1.0	1370/71853 (73.9)	1.0	1.0
Episiotomy	688/863 (79.7)	0.72 (0.61–0.86)	0.73 (0.59–0.89)	194/275 (70.6)	0.84 (0.64–1.12)	0.81 (0.58–1.12)
Involved in decision‐making						
No epidural	5561/6806 (81.7)	1.0	1.0	396/565 (70.1)	1.0	1.0
Epidural	7374/8699 (84.8)	1.25 (1.14–1.36)	1.33 (1.21–1.47)	1095/1602 (68.4)	0.92 (0.75–1.14)	1.05 (0.82–1.34)
No oxytocin	6145/7283 (84.4)	1.0	1.0	105/160 (65.6)	1.0	1.0
Oxytocin	6790/8222 (82.6)	0.88 (0.81–0.96)	0.87 (0.79–0.96)	1386/2007 (69.1)	1.17 (0.83–1.64)	1.21 (0.81–1.83)
No episiotomy	11 841/14131 (83.8)	1.0	1.0	1279/1834 (69.7)	1.0	1.0
Episiotomy	664/853 (77.8)	0.68 (0.57–0.80)	0.60 (0.50–0.73)	174/272 (64.0)	0.77 (0.59–1.01)	0.81 (0.59–1.11)

*Note:* Adjusted for age, BMI, country of birth, level of education, mental illness, positive self‐assessed health before pregnancy, fear of birth, pre‐pregnancy comorbidity, pregnancy comorbidity, gestational age at birth, and hospital size.

Of the analyses conducted among women with instrumental births, only one reached statistical significance, though its clinical relevance was limited (Table 3). Consequently, we chose to further analyse combinations of interventions and respectful care exclusively among women with spontaneous vaginal births. Additionally, a sensitivity analysis was performed since experiencing an adverse outcome may influence a woman's perception of respectful care. We chose to conduct this analysis for postpartum haemorrhage (PPH, ≥ 1000 mL), as severe postpartum bleeding can be perceived by women as a life‐threatening event and may also influence their perception of the aspects examined in this study, primarily support and information [30]. Results remained largely unchanged when women with PPH were excluded from the analysis (Table S3).

### 3.2 Association Between Combination of Interventions and Respectful Care

**FIGURE 1:**
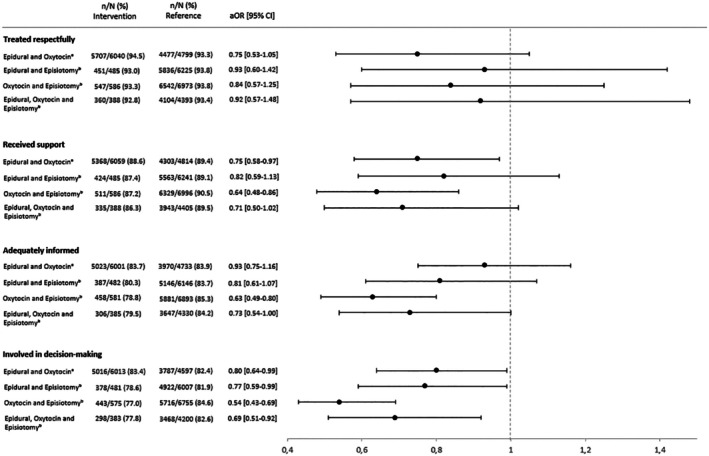
Adjusted odds ratios (aOR) with 95% confidence intervals (CI) for women's perceptions of respect, support, information, and involvement in decision‐making by use of medical interventions. Primiparous women with spontaneous labour onset and spontaneous vaginal births, Sweden, 2022–2023. ^a^Model A: including an interaction term (oxytocin augmentation#epidural analgesia). Adjusted for age, BMI, country of birth, level of education, mental illness, positive self‐assessed health before pregnancy, fear of birth, pre‐pregnancy comorbidity, pregnancy comorbidity, gestational age at birth, and hospital size. ^b^Model B: adjusted for age, BMI, country of birth, level of education, mental illness, positive self‐assessed health before pregnancy, fear of birth, pre‐pregnancy comorbidity, pregnancy comorbidity, gestational age at birth, and hospital size.

For women with spontaneous vaginal births, receiving two or more interventions did not significantly affect their perceptions of being treated with respect. There was a statistically significant difference associated with the use of both epidural and oxytocin in relation to the perception of being supported by staff (88.6% vs. 89.4%; aOR 0.75, 95% CI 0.58–0.97). However, when interpreted in clinical practice, this finding is unlikely to represent a meaningful difference (Figure 1). In contrast, the combination of oxytocin and episiotomy was associated with the lowest adjusted odds for reporting adequate support (87.2% vs. 90.5%; aOR 0.64, 95% CI 0.48–0.86), information (78.8% vs. 85.3%; aOR 0.63, 95% CI 0.49–0.80), and for being involved in decision‐making (77.0% vs. 84.6%; aOR 0.54, 95% CI 0.43–0.69). Notably, the largest absolute difference in proportions, with 7.6 percentage points, was observed for involvement in decision‐making (Figure 1). When women experienced all three interventions; that is, epidural, oxytocin, and episiotomy, the odds of reporting involvement in decision‐making were significantly lower compared to women who received no interventions (77.8% vs. 82.6%; aOR 0.69, 95% CI 0.51–0.92) (Figure 1).

### 3.3 Main Findings

This national survey‐based cohort study found that most primiparous women experienced respectful care during labour and birth. Only a minority of the respondents felt they were not treated with respect or inadequately supported. However, approximately one‐sixth of the women reported feeling inadequately informed, while nearly one‐fifth felt they were insufficiently involved in the decision‐making process. Women with spontaneous vaginal births who were subjected to an episiotomy were less likely to report being involved in decision‐making. The combined use of oxytocin and episiotomy was associated with decreased odds of respectful care, particularly regarding involvement in decision‐making.

### 3.4 Strengths and Limitations

The main strength of the study is the combination of high‐quality national register data with survey responses on women's perceptions of care during labour and birth. The prospectively collected register data from the SPR, including detailed sociodemographic, maternal health, and pregnancy‐related variables, allowed for adjustments of multiple confounders. The SPR further includes labour data, which meant that the interventions under study, that is, epidural, oxytocin, and episiotomy were obtained from the register and not self‐reported.

Additionally, the large sample size enabled us to analyse how these interventions affected experiences both individually and in combination. It further enabled stratification by mode of birth, which is known to influence the childbirth experience [31, 32], and may also influence the experience of respectful care. However, due to the large sample size, some results with very small differences reached statistical significance, even though they are unlikely to be of clinical relevance.

The NPS‐8 is distributed 8 weeks postpartum, which is a common time point for measuring childbirth experience and satisfaction with care [33]. There is insufficient evidence on which to base conclusions about the impact of the timing of satisfaction assessment during childbirth care. Previous research does not unanimously show whether, or how, women's childbirth experiences change over time [33, 34], but the overall perception of childbirth has been shown to become more negative with time [35]. Whether this applies to the experience of respectful care has, to our knowledge, not been studied and should be the subject of future research.

Another limitation is the number of missing responses for the outcome variables under study. The higher proportion of missing responses is attributable to the inclusion of surveys that were partially and not only entirely completed. Furthermore, consistent with other studies using a cross‐sectional design [1, 36], non‐responders to the NPS‐8 were more likely to be younger, born outside the Nordic countries, and have lower educational attainment than responders, which are all factors previously associated with negative childbirth experiences [36, 37]. Consequently, the findings may not be fully generalisable to the broader population of Swedish primiparous women, although intervention rates did not differ between responders and non‐responders. Finally, while adjustments were made for multiple confounders, the possibility of residual confounding cannot be ruled out.

### 3.5 Interpretation

The present study found that the vast majority of primiparous women reported being treated with respect during labour and birth. However, a significantly lower proportion of women who underwent instrumental birth reported experiencing respectful care. This finding aligns with previous research on childbirth experiences, where the mode of birth has consistently been identified as one of the strongest predictors of a negative experience [38, 39, 40]. Notably, a third of the women who had an instrumental birth and 15% of those with a spontaneous vaginal birth indicated that they did not feel adequately involved in decision‐making. This is a concerning finding that warrants further investigation, particularly given that the study population was limited to women with spontaneous onset of labour, representing a predominantly low‐risk group.

Although statistically significant differences were observed for oxytocin and epidural, the absolute differences in proportions were small. From a clinical perspective, our findings therefore indicate that the use of these interventions, whether administered individually or in combination, does not substantially influence women's perceptions of support, information, or involvement in decision‐making during childbirth. This contrasts with previous studies that have linked medical interventions to an increased risk of experiencing disrespectful care [15, 16, 17]. Furthermore, a recent study by Brüggemann et al. (2024) found that women who received a high cumulative oxytocin dose during labour were more likely to report an overall negative childbirth experience [41]. One possible explanation for our results not aligning with previous research on childbirth experience is that support, information, and involvement in decision‐making, although vital parts of the childbirth experience, do not encompass all of its aspects [42]. As the childbirth experience is multidimensional, negative experiences in one dimension may also be mitigated by positive experiences in others [43].

Nevertheless, our findings indicate that there is an association between the use of epidural or oxytocin and women's perceptions of being supported, receiving information, and being involved in decision‐making at the population level. Given these associations, both epidural analgesia and oxytocin remain important to study: epidural since it often marks the initiation of subsequent interventions [44, 45], and oxytocin due to its potency as a high‐risk medication known to carry potential adverse effects, including uterine hyperstimulation, foetal distress, and an increased risk of emergency caesarean section [46, 47, 48].

The way questions are formulated in surveys can influence how respondents interpret and answer them. In the present study, four items reflecting different aspects of respectful care were used. Although all questions in the NPS‐8 were developed with input from both healthcare professionals and women, their psychometric properties have not been evaluated. In contrast to validated scales, which comprise multiple items designed to measure an underlying construct, single‐item measures are more vulnerable to random measurement error and do not permit assessment of internal consistency reliability. Further research using validated scales is therefore required to investigate whether interventions affect women's experiences of respectful care [49, 50].

Our results further revealed an association between episiotomy and experiences of respectful care, negatively affecting women's perceptions of support, information, and involvement in decision‐making. Episiotomy has previously been strongly associated with disrespectful and abusive care [2, 4, 16, 51], and when performed without consent, it has been considered obstetric violence [52, 53]. Data from the US survey “Giving Voice to Mothers” revealed that most women felt they had no choice in undergoing this procedure [1]. A recently published Swedish study further showed that women who were subjected to an episiotomy reported the lowest rate of informed consent among interventions performed during the second stage of labour [54]. Given the restrictive use of episiotomy in Sweden [18], its association with respectful care might have been expected to be less pronounced. However, since episiotomies in Sweden are uncommon in spontaneous vaginal births, they may be more likely to occur in urgent situations where medical necessity is prioritised over obtaining informed consent. This highlights the need for improved communication and involvement in decision‐making, even in acute situations.

## 4 Conclusion

This study shows that the majority of primiparous women with spontaneous labour onset experienced respectful care during labour and birth. Among women who gave birth spontaneously, the odds of reporting involvement in decision‐making were lower for women with an episiotomy or who were both augmented with oxytocin and subjected to an episiotomy. Continued efforts are needed to implement and evaluate interventions and care processes that promote involvement in decision‐making during labour and birth.

## Author Contributions

Karin Johnson and Malin Edqvist designed the study with input from LE, KJ, and SS Malin Edqvist was responsible for the acquisition of data. Karin Johnson performed the analyses, curated the tables and figures, and wrote the first draft of the manuscript. All authors were involved in interpreting the results, contributed to the manuscript, and approved the final version.

## Ethics Statement

The study was approved by the Swedish Ethical Review Authority at Karolinska no 2020‐02500 on October 10, 2020, and amendment 2020‐06872 on January 4, 2021. All data management and analyses were conducted on de‐identified data where the personal identification numbers have been replaced with pseudo‐anonymous serial numbers. No informed consent was needed for the register data. For the NPS‐8 survey, all pregnant women are informed that their responses are recorded in the SPR and can be used for research purposes. They are further informed of their right to withdraw their consent and survey responses at any time, including the option to opt‐out from the SPR.

## Conflicts of Interest

The authors declare no conflicts of interest.

## Supporting information


**Figure S1:** bjo18329‐sup‐0001‐FigureS1.docx.

**Data S1:** bjo18329‐sup‐0002‐DataS1.docx.

**Table S1:** bjo18329‐sup‐0003‐TableS1.docx.

**Table S2:** bjo18329‐sup‐0004‐TableS2.docx.

**Table S3:** bjo18329‐sup‐0005‐TableS3.docx.

## Data Availability

The data supporting the findings of this study are available on request from the corresponding author. The data are not publicly available due to privacy and ethical restrictions.

## References

[1] S. Vedam , K. Stoll , T. K. Taiwo , et al., “The Giving Voice to Mothers Study: Inequity and Mistreatment During Pregnancy and Childbirth in the United States,” Reproductive Health 16 (2019): 77. 31182118 10.1186/s12978-019-0729-2 PMC6558766

[2] M. S. G. van der Pijl , C. J. M. Verhoeven , R. Verweij , et al., “Disrespect and Abuse During Labour and Birth Amongst 12,239 Women in The Netherlands: A National Survey,” Reproductive Health 19 (2022): 160. 35804419 10.1186/s12978-022-01460-4 PMC9266084

[3] L. K. Fraser , N. Cano‐Ibáñez , C. Amezcua‐Prieto , K. S. Khan , R. F. Lamont , and J. S. Jørgensen , “Prevalence of Obstetric Violence in High‐Income Countries: A Systematic Review of Mixed Studies and Meta‐Analysis of Quantitative Studies,” Acta Obstetricia et Gynecologica Scandinavica 104 (2024): 13–28. 39278647 10.1111/aogs.14962 PMC11683541

[4] M. S. G. van der Pijl , M. H. Hollander , T. van der Linden , et al., “Left Powerless: A Qualitative Social Media Content Analysis of the Dutch #Breakthesilence Campaign on Negative and Traumatic Experiences of Labour and Birth,” PLoS One 15 (2020): e0233114. 32396552 10.1371/journal.pone.0233114 PMC7217465

[5] J. Slomian , G. Honvo , P. Emonts , J. Y. Reginster , and O. Bruyère , “Consequences of Maternal Postpartum Depression: A Systematic Review of Maternal and Infant Outcomes,” Women's Health (London, England) 15 (2019): 1745506519844044. 10.1177/1745506519844044 PMC6492376 31035856

[6] J. M. Martínez‐Galiano , A. Rubio‐Alvárez , A. Ballesta‐Castillejos , I. Ortiz‐Esquinas , M. Donate‐Manzanares , and A. Hernández‐Martínez , “Risk of Suicide and Postpartum Depression in Women Who Feel They Were Treated Inadequately During Childbirth,” Women and Birth 38 (2025): 101858. 39752767 10.1016/j.wombi.2024.101858

[7] A. P. Betran , J. Ye , A. B. Moller , J. P. Souza , and J. Zhang , “Trends and Projections of Caesarean Section Rates: Global and Regional Estimates,” BMJ Global Health 6 (2021): e005671. 10.1136/bmjgh-2021-005671 PMC8208001 34130991

[8] O. T. Oladapo , Ö. Tunçalp , M. Bonet , et al., “WHO Model of Intrapartum Care for a Positive Childbirth Experience: Transforming Care of Women and Babies for Improved Health and Wellbeing,” BJOG: An International Journal of Obstetrics and Gynaecology 125 (2018): 918–922. 29637727 10.1111/1471-0528.15237 PMC6033015

[9] H. L. Shuman , A. M. Grupp , L. A. Robb , et al., “Approaches and Geographical Locations of Respectful Maternity Care Research: A Scoping Review,” PLoS One 18 (2023): e0290434. 37616299 10.1371/journal.pone.0290434 PMC10449213

[10] I. Hildingsson , “Women's Birth Expectations, Are They Fulfilled? Findings From a Longitudinal Swedish Cohort Study,” Women and Birth 28 (2015): e7–e13. 25700792 10.1016/j.wombi.2015.01.011

[11] A. Annborn and H. R. Finnbogadóttir , “Obstetric Violence a Qualitative Interview Study,” Midwifery 105 (2022): 103212. 34872035 10.1016/j.midw.2021.103212

[12] N. Malhotra , C. M. Jevitt , K. Stoll , W. Phillips‐Beck , S. Vedam , and the RESPCCT Study Team , “Weight‐Based Disparities in Perinatal Care: Quantitative Findings of Respect, Autonomy, Mistreatment, and Body Mass Index in a National Canadian Survey,” BMC Pregnancy and Childbirth 24 (2024): 737. 39516762 10.1186/s12884-024-06928-8 PMC11549742

[13] L. B. Attanasio , K. B. Kozhimannil , and K. H. Kjerulff , “Factors Influencing Women's Perceptions of Shared Decision Making During Labor and Delivery: Results From a Large‐Scale Cohort Study of First Childbirth,” Patient Education and Counseling 101 (2018): 1130–1136. 29339041 10.1016/j.pec.2018.01.002 PMC5977392

[14] M. Lukasse , A. M. Schroll , H. Karro , et al., “Prevalence of Experienced Abuse in Healthcare and Associated Obstetric Characteristics in Six European Countries,” Acta Obstetricia et Gynecologica Scandinavica 94 (2015): 508–517. 25627169 10.1111/aogs.12593

[15] D. R. Leijerzapf , M. S. G. van der Pijl , M. H. Hollander , E. Kingma , A. de Jonge , and C. J. M. Verhoeven , “Experienced Disrespect & Abuse During Childbirth and Associated Birth Characteristics: A Cross‐Sectional Survey in The Netherlands,” BMC Pregnancy and Childbirth 24 (2024): 170. 38424515 10.1186/s12884-024-06360-y PMC10905902

[16] C. Liu , K. Underhill , J. J. Aubey , G. Samari , H. L. Allen , and J. R. Daw , “Disparities in Mistreatment During Childbirth,” JAMA Network Open 7 (2024): e244873. 38573636 10.1001/jamanetworkopen.2024.4873 PMC11192180

[17] J. M. Martínez‐Galiano , S. Martinez‐Vazquez , J. Rodríguez‐Almagro , and A. Hernández‐Martinez , “The Magnitude of the Problem of Obstetric Violence and Its Associated Factors: A Cross‐Sectional Study,” Women and Birth 34 (2021): e526–e536. 33082123 10.1016/j.wombi.2020.10.002

[18] Y. C. P. Skogsdal , C. Elvander , C. Hed , et al., “The Annual Report of the Swedish Pregnancy Register 2023,” 2024. The Swedish Pregnancy Register, www.graviditetsregistret.se.

[19] A. E. Seijmonsbergen‐Schermers , T. van den Akker , E. Rydahl , et al., “Variations in Use of Childbirth Interventions in 13 High‐Income Countries: A Multinational Cross‐Sectional Study,” PLoS Medicine 17 (2020): e1003103. 32442207 10.1371/journal.pmed.1003103 PMC7244098

[20] S. Miller , E. Abalos , M. Chamillard , et al., “Beyond Too Little, Too Late and Too Much, Too Soon: A Pathway Towards Evidence‐Based, Respectful Maternity Care Worldwide,” Lancet 388 (2016): 2176–2192. 27642019 10.1016/S0140-6736(16)31472-6

[21] M. Anim‐Somuah , R. M. Smyth , A. M. Cyna , and A. Cuthbert , “Epidural Versus Non‐Epidural or no Analgesia for Pain Management in Labour,” Cochrane Database of Systematic Reviews 5 (2018): CD000331. 29781504 10.1002/14651858.CD000331.pub4 PMC6494646

[22] R. Lu , L. Rong , L. Ye , Y. Xu , and H. Wu , “Effects of Epidural Analgesia on Intrapartum Maternal Fever and Maternal Outcomes: An Updated Systematic Review and Meta‐Analysis,” Journal of Maternal‐Fetal & Neonatal Medicine 37 (2024): 2357168. 38812361 10.1080/14767058.2024.2357168

[23] J. Leinweber , Y. Fontein‐Kuipers , S. I. Karlsdottir , et al., “Developing a Woman‐Centered, Inclusive Definition of Positive Childbirth Experiences: A Discussion Paper,” Birth 50 (2023): 362–383. 35790019 10.1111/birt.12666

[24] O. Stephansson , K. Petersson , C. Björk , P. Conner , and A. K. Wikström , “The Swedish Pregnancy Register–For Quality of Care Improvement and Research,” Acta Obstetricia et Gynecologica Scandinavica 97 (2018): 466–476. 29172245 10.1111/aogs.13266 PMC5873375

[25] Sveriges Kommuner och Regioner , “The Swedish Association of Local Authorities and Regions (SALAR): The Regions' Efforts for Women's Health,” 2023 [cited Jan 10 2025], pp 138, https://skr.se/download/18.3ecbf48018722d64006266c4/1680182194171/Regioneras_insatser_kvinnors_halsa_2022.pdf.

[26] M. Ahlberg , “PROM and PREM Questions in the Pregnancy Register–A Pilot Study,” 2015, The Swedish Pregnancy Register, Stockholm, Sweden.

[27] M. J. Guittier , C. Cedraschi , N. Jamei , M. Boulvain , and F. Guillemin , “Impact of Mode of Delivery on the Birth Experience in First‐Time Mothers: A Qualitative Study,” BMC Pregnancy and Childbirth 1, no. 14 (2014): 254. 10.1186/1471-2393-14-254 PMC4132899 25080994

[28] M. Chabbert , D. Panagiotou , and J. Wendland , “Predictive Factors of Women's Subjective Perception of Childbirth Experience: A Systematic Review of the Literature,” Journal of Reproductive and Infant Psychology 39 (2021): 43–66. 32475156 10.1080/02646838.2020.1748582

[29] N. Shrestha , “Detecting Multicollinearity in Regression Analysis,” American Journal of Applied Mathematics and Statistics 8 (2020): 39–42.

[30] J. F. Thompson , J. B. Ford , C. H. Raynes‐Greenow , C. L. Roberts , and D. A. Ellwood , “Women's Experiences of Care and Their Concerns and Needs Following a Significant Primary Postpartum Hemorrhage,” Birth 38 (2011): 327–335. 22112333 10.1111/j.1523-536X.2011.00491.x

[31] A. Smarandache , T. H. Kim , Y. Bohr , and H. Tamim , “Predictors of a Negative Labour and Birth Experience Based on a National Survey of Canadian Women,” BMC Pregnancy and Childbirth 16 (2016): 114. 27193995 10.1186/s12884-016-0903-2 PMC4870779

[32] I. Wiklund , G. Edman , E. L. Ryding , and E. Andolf , “Expectation and Experiences of Childbirth in Primiparae With Caesarean Section,” BJOG: An International Journal of Obstetrics and Gynaecology 115 (2008): 324–331. 18190368 10.1111/j.1471-0528.2007.01564.x

[33] H. Nilvér , C. Begley , and M. Berg , “Measuring Women's Childbirth Experiences: A Systematic Review for Identification and Analysis of Validated Instruments,” BMC Pregnancy and Childbirth 17 (2017): 203. 28662645 10.1186/s12884-017-1356-y PMC5492707

[34] E. D. Hodnett , “Pain and Women's Satisfaction With the Experience of Childbirth: A Systematic Review,” American Journal of Obstetrics and Gynecology 186 (2002): S160–S172. 12011880 10.1067/mob.2002.121141

[35] K. Lyngbye , D. Melgaard , V. Lindblad , et al., “Do Women's Perceptions of Their Childbirth Experiences Change Over Time? A Six‐Week Follow‐Up Study in a Danish Population,” Midwifery 113 (2022): 103429. 35901608 10.1016/j.midw.2022.103429

[36] M. A. Bohren , H. Mehrtash , B. Fawole , et al., “How Women Are Treated During Facility‐Based Childbirth in Four Countries: A Cross‐Sectional Study With Labour Observations and Community‐Based Surveys,” Lancet 394 (2019): 1750–1763. 31604660 10.1016/S0140-6736(19)31992-0 PMC6853169

[37] F. Fair , L. Raben , H. Watson , et al., “Migrant Women's Experiences of Pregnancy, Childbirth and Maternity Care in European Countries: A Systematic Review,” PLoS One 15 (2020): e0228378. 32045416 10.1371/journal.pone.0228378 PMC7012401

[38] T. A. S. Muhamed , V. Angelini , L. Viluma , H. Keedle , and L. L. Peters , “Negative Childbirth Experience in Dutch Women: A Socio‐Ecological Analysis of Individual, Interpersonal, and Organisational Factors From the Birth Experience Study,” Heliyon 11 (2025): e41254. 39958747 10.1016/j.heliyon.2024.e41254 PMC11825256

[39] P. Kempe and M. Vikström‐Bolin , “Women's Satisfaction With the Birthing Experience in Relation to Duration of Labour, Obstetric Interventions and Mode of Birth,” European Journal of Obstetrics, Gynecology, and Reproductive Biology 246 (2020): 156–159. 32028143 10.1016/j.ejogrb.2020.01.041

[40] A. Dencker , C. Nilsson , C. Begley , et al., “Causes and Outcomes in Studies of Fear of Childbirth: A Systematic Review,” Women and Birth 32 (2019): 99–111. 30115515 10.1016/j.wombi.2018.07.004

[41] C. Brüggemann , S. Carlhäll , H. Grundström , A. Ramö Isgren , and M. Blomberg , “Cumulative Oxytocin Dose in Spontaneous Labour–Adverse Postpartum Outcomes, Childbirth Experience, and Breastfeeding,” European Journal of Obstetrics, Gynecology, and Reproductive Biology 295 (2024): 98–103. 38350309 10.1016/j.ejogrb.2024.01.040

[42] F. Viirman , S. Hesselman , A. K. Wikström , et al., “Negative Childbirth Experience–What Matters Most? A Register‐Based Study of Risk Factors in Three Time Periods During Pregnancy,” Sexual & Reproductive Healthcare 34 (2022): 100779. 36152452 10.1016/j.srhc.2022.100779

[43] A. Volkert , L. Bach , C. Hagenbeck , et al., “Obstetric Interventions' Effects on the Birthing Experience,” BMC Pregnancy and Childbirth 24 (2024): 508. 39068395 10.1186/s12884-024-06626-5 PMC11283698

[44] A. Petersen , U. Poetter , C. Michelsen , and M. M. Gross , “The Sequence of Intrapartum Interventions: A Descriptive Approach to the Cascade of Interventions,” Archives of Gynecology and Obstetrics 288 (2013): 245–254. 23417149 10.1007/s00404-013-2737-8

[45] A. Westergren , K. Edin , M. Lindkvist , and M. Christianson , “Exploring the Medicalisation of Childbirth Through Women's Preferences for and Use of Pain Relief,” Women and Birth 34 (2021): e118–e127. 32094035 10.1016/j.wombi.2020.02.009

[46] Institute for Safe Medication Practices (ISMP) , “ISMP's List of High‐Alert Medications,” 2008, www.ismp.org.

[47] C. A. Grotegut , M. J. Paglia , L. N. Johnson , B. Thames , and A. H. James , “Oxytocin Exposure During Labor Among Women With Postpartum Hemorrhage Secondary to Uterine Atony,” American Journal of Obstetrics and Gynecology 204 (2011): 56.e1‐56.e566. 10.1016/j.ajog.2010.08.023 PMC3018152 21047614

[48] M. Jonsson , S. Nordén‐Lindeberg , I. Ostlund , and U. Hanson , “Acidemia at Birth, Related to Obstetric Characteristics and to Oxytocin Use, During the Last Two Hours of Labor,” Acta Obstetricia et Gynecologica Scandinavica 87 (2008): 745–750. 18607817 10.1080/00016340802220352

[49] S. Vedam , K. Stoll , K. Martin , et al., “The Mother's Autonomy in Decision Making (MADM) Scale: Patient‐Led Development and Psychometric Testing of a New Instrument to Evaluate Experience of Maternity Care,” PLoS One 12 (2017): e0171804. 28231285 10.1371/journal.pone.0171804 PMC5322919

[50] S. Vedam , K. Stoll , N. Rubashkin , et al., “The Mothers on Respect (MOR) Index: Measuring Quality, Safety, and Human Rights in Childbirth,” SSM ‐ Population Health 3 (2017): 201–210. 29349217 10.1016/j.ssmph.2017.01.005 PMC5768993

[51] M. van der Pijl , C. Verhoeven , M. Hollander , A. de Jonge , and E. Kingma , “The Ethics of Consent During Labour and Birth: Episiotomies,” Journal of Medical Ethics 49 (2023): 611–617. 36717252 10.1136/jme-2022-108601 PMC10511989

[52] M. Jacques , A. A. Chantry , A. Evrard , N. Lelong , and C. Le Ray , “Consent for Interventions During Childbirth: A National Population‐Based Study,” International Journal of Gynecology & Obstetrics 168 (2024): 333–342. 39092580 10.1002/ijgo.15830 PMC11649875

[53] T. Djanogly , J. Nicholls , M. Whitten , and A. Lanceley , “Choice in Episiotomy–Fact or Fantasy: A Qualitative Study of Women's Experiences of the Consent Process,” BMC Pregnancy and Childbirth 22 (2022): 139. 35189846 10.1186/s12884-022-04475-8 PMC8862370

[54] M. Edqvist , I. Rådestad , I. Lundgren , M. Mollberg , and H. Lindgren , “Practices Used by Midwives During the Second Stage of Labor to Facilitate Birth–Are They Related to Perineal Trauma?,” Sexual & Reproductive Healthcare 15 (2018): 18–22. 29389495 10.1016/j.srhc.2017.11.003

