# Supplementary material for: Medical Interventions and Women's Perceptions of Respectful Intrapartum Care: A National Survey‐Based Cohort Study
Source: BJOG. 2025 Aug 11;132(12):1844–55. doi: 10.1111/1471-0528.18329 (PMC12501740; doi:10.1111/1471-0528.18329)
Supplement: Supplementary file 1 — Figure S1: bjo18329‐sup‐0001‐FigureS1.docx. [file BJO-132-1844-s002.docx]

**Figure S1. Flow diagram of the study population**

Nulliparous women giving birth between January 1^st^ 2022 – December 31^st^ 2023

N= 34 314

Non-responders to the 8-week

Pregnancy Survey

N= 13 748

Population

N= 34 111

Duplicates 8-week survey

N= 203

Instrumental birth

N= 2317

Spontaneous vaginal birth

N= 16 677

Final study population

N= 18 994

Non-responders to the first four items in the 8-week Pregnancy Survey

N= 1369

Population

N= 20 363
