# Supplementary material for: Medical Interventions and Women's Perceptions of Respectful Intrapartum Care: A National Survey‐Based Cohort Study
Source: BJOG. 2025 Aug 11;132(12):1844–55. doi: 10.1111/1471-0528.18329 (PMC12501740; doi:10.1111/1471-0528.18329)
Supplement: Supplementary file 2 — Data S1: bjo18329‐sup‐0002‐DataS1.docx. [file BJO-132-1844-s005.docx]

# The Swedish National Pregnancy Survey no. 2 (NPS-8)

## Your health

**1. How do you perceive your general state of health in the final three months of pregnancy?**

☐ 1 Very bad ☐ 2 ☐ 3 ☐ 4 ☐ 5 Very good ☐ Cannot / do not wish to answer

## Antenatal care

**2. Was it easy to get in touch with the midwife at the antenatal care centre?**

☐ 1 No, not at all ☐ 2 ☐ 3 ☐ 4 ☐ 5 Yes, completely ☐ Cannot / do not wish to answer

**3. Were you able to ask the midwife any questions you had?**

☐ 1 No, not at all ☐ 2 ☐ 3 ☐ 4 ☐ 5 Yes, completely ☐ Cannot / do not wish to answer

**4. During your pregnancy, were you involved in planning and decision-making to the extent you desired?**

☐ 1 No, not at all ☐ 2 ☐ 3 ☐ 4 ☐ 5 Yes, completely ☐ Cannot / do not wish to answer

**5. Did you feel safe and comfortable with the midwife at the antenatal care centre?**

☐ 1 No, not at all ☐ 2 ☐ 3 ☐ 4 ☐ 5 Yes, completely ☐ Cannot / do not wish to answer

**6. Were you able to get support from the midwife when you felt you needed it? (e.g., if you felt worried, frightened, had anxiety or similar)**

☐ 1 No, not at all ☐ 2 ☐ 3 ☐ 4 ☐ 5 Yes, completely ☐ Cannot / do not wish to answer ☐ Did not need support

**7. Did the midwife at the antenatal care centre involve your partner/next of kin as much as you wanted?**

☐ 1 No, not at all ☐ 2 ☐ 3 ☐ 4 ☐ 5 Yes, completely ☐ Cannot / do not wish to answer ☐ Not applicable

**8. If you attended any antenatal group meetings – did you find them valuable?**

☐ 1 No, not at all ☐ 2 ☐ 3 ☐ 4 ☐ 5 Yes, completely ☐ Cannot / do not wish to answer ☐ Did not attend group meetings

**9. Do you feel that the antenatal care professionals coordinated your other healthcare contacts to the extent needed?**

☐ 1 No, not at all ☐ 2 ☐ 3 ☐ 4 ☐ 5 Yes, completely ☐ Cannot / do not wish to answer ☐ Not applicable

**10. Would you recommend your antenatal centre to someone else who is pregnant?**

☐ 1 No, not at all ☐ 2 ☐ 3 ☐ 4 ☐ 5 Yes, completely ☐ Cannot / do not wish to answer

**11. Do you feel that the antenatal care services met your needs**?

☐ 1 No, not at all ☐ 2 ☐ 3 ☐ 4 ☐ 5 Yes, completely ☐ Cannot / do not wish to answer

## Labour and birth

**12. How did you give birth?**

☐ Vaginal birth ☐ Planned caesarean section ☐ Emergency caesarean section ☐ Cannot / do not wish to answer

**13. Was this your first delivery, or have you given birth before?**

☐ It was my first child ☐ I have given birth before ☐ Cannot / do not wish to answer

**14. How did you perceive the birth?**

☐ 1 Worst imaginable ☐ 2 ☐ 3 ☐ 4 ☐ 5 ☐ 6 ☐ 7 ☐ 8 ☐ 9 ☐ 10 Best imaginable ☐ Cannot / do not wish to answer

**15a. Were you able to give birth in the location you wanted?**

☐ Yes → Go to question 16 ☐ No ☐ Cannot / do not wish to answer → Go to question 16

**15b. Why were you unable to give birth in the location you wanted?**

☐ Referred elsewhere due to lack of space ☐ Referred for medical reasons ☐ Referred but don't know why ☐ Other: ___________ ☐ Cannot / do not wish to answer

**16. During labour and birth, were you involved in planning and decision-making to the extent you desired?**

☐ 1 No, not at all ☐ 2 ☐ 3 ☐ 4 ☐ 5 Yes, completely ☐ Cannot / do not wish to answer ☐ Not applicable

**17. Did you get the pain relief you needed when you gave birth?**

☐ 1 No, not at all ☐ 2 ☐ 3 ☐ 4 ☐ 5 Yes, completely ☐ Cannot / do not wish to answer ☐ Not applicable

**18. During labour and birth, did you receive support from the caregivers to the extent you desired?**

☐ 1 No, not at all ☐ 2 ☐ 3 ☐ 4 ☐ 5 Yes, completely ☐ Cannot / do not wish to answer ☐ Not applicable

**19. Did the midwife spend as much time in the delivery room as you wanted?**

☐ 1 No, not at all ☐ 2 ☐ 3 ☐ 4 ☐ 5 Yes, completely ☐ Cannot / do not wish to answer ☐ Not applicable

**20. Did you feel that the healthcare professionals cooperated well?**

☐ 1 No, not at all ☐ 2 ☐ 3 ☐ 4 ☐ 5 Yes, completely ☐ Cannot / do not wish to answer

**21. During labour and birth, did the caregivers treat you with respect?**

☐ 1 No, not at all ☐ 2 ☐ 3 ☐ 4 ☐ 5 Yes, completely ☐ Cannot / do not wish to answer

**22. Did the healthcare professionals at the maternity unit involve your partner/next of kin as much as you wanted?**

☐ 1 No, not at all ☐ 2 ☐ 3 ☐ 4 ☐ 5 Yes, completely ☐ Cannot / do not wish to answer ☐ Not applicable

**23. During labour and birth, did you receive enough information?**

☐ 1 No, not at all ☐ 2 ☐ 3 ☐ 4 ☐ 5 Yes, completely ☐ Cannot / do not wish to answer

**24. Did you feel safe and comfortable with the care you received when giving birth?**

☐ 1 No, not at all ☐ 2 ☐ 3 ☐ 4 ☐ 5 Yes, completely ☐ Cannot / do not wish to answer

**25. Were you allowed to hold your baby skin-to-skin directly after the birth, until the baby latched on and/or fell asleep?**

☐ Yes ☐ Yes, partly ☐ No ☐ Don't remember ☐ Cannot / do not wish to answer

**26. Did you receive as much support as you wanted with the first breastfeeding?**

☐ 1 No, not at all ☐ 2 ☐ 3 ☐ 4 ☐ 5 Yes, completely ☐ Cannot / do not wish to answer ☐ Not applicable

**27. Would you recommend someone else to give birth in the same maternity unit?**

☐ 1 No, not at all ☐ 2 ☐ 3 ☐ 4 ☐ 5 Yes, completely ☐ Cannot / do not wish to answer

**28. Were you allowed to stay for as long as you needed after the birth?**

☐ Yes ☐ No, I would have liked to stay longer ☐ No, I would have liked to leave earlier ☐ Cannot / do not wish to answer

**29. Was your partner/next of kin given the opportunity to stay after the birth?**

☐ Yes ☐ No, but I would have liked that ☐ No, it wasn’t necessary ☐ Not applicable ☐ Cannot / do not wish to answer

**30. Were you given the opportunity to have a postnatal or discharge conversation with your midwife or doctor before you went home?**

☐ Yes ☐ No, but I would have liked that ☐ No, it wasn’t necessary ☐ Cannot / do not wish to answer

**31. Did you receive the information you needed on what to expect regarding your recovery/the time after the birth?**

☐ 1 No, not at all ☐ 2 ☐ 3 ☐ 4 ☐ 5 Yes, completely ☐ Cannot / do not wish to answer

**32. Did the healthcare professionals involve you as much as you wanted in the planning and decisions regarding your postnatal care?**

☐ 1 No, not at all ☐ 2 ☐ 3 ☐ 4 ☐ 5 Yes, completely ☐ Cannot / do not wish to answer ☐ Not applicable

**33. Did the healthcare professionals involve your partner/next of kin as much as you wanted after the birth?**

☐ 1 No, not at all ☐ 2 ☐ 3 ☐ 4 ☐ 5 Yes, completely ☐ Cannot / do not wish to answer ☐ Not applicable

## Your health

**34. What is your assessment of your general state of health right now?**

☐ 1 Very bad ☐ 2 ☐ 3 ☐ 4 ☐ 5 Very good ☐ Cannot / do not wish to answer

**35. Do you have medical issues that you didn’t expect or postnatal complications?**

☐ Yes, severe/serious ☐ Yes, mild ☐ No ☐ Cannot / do not wish to answer

**36. Do you have medical issues or pain in the genital area after the birth?**

☐ Yes ☐ No ☐ Prefer not to answer

**37. Do you have, or have you had, difficulties emptying your bladder and needed treatment for this after the birth?**

☐ Yes ☐ No ☐ Prefer not to answer

**38. Do you suffer from a leaky bladder or the unintentional passing of urine?**

☐ Never ☐ Almost never ☐ 1–3 times per month ☐ 1–3 times per week ☐ Daily ☐ Prefer not to answer

**39a. Do you find it difficult to control your faeces or flatulence?**

☐ No → Go to question 40 ☐ Yes ☐ Prefer not to answer → Go to question 40

**39b. Do you ever pass wind even if it’s inappropriate?**

☐ Never ☐ Almost never ☐ 1–3 times per month ☐ 1–3 times per week ☐ Daily ☐ Prefer not to answer

**39c. Do you suffer from leakages of loose faeces?**

☐ Never ☐ Almost never ☐ 1–3 times per month ☐ 1–3 times per week ☐ Daily ☐ Prefer not to answer

**39d. Do you suffer from leakages of solid faeces?**

☐ Never ☐ Almost never ☐ 1–3 times per month ☐ 1–3 times per week ☐ Daily ☐ Prefer not to answer

**39e. Do you use incontinence products due to bowel leakage?**

☐ Never ☐ Almost never ☐ 1–3 times per month ☐ 1–3 times per week ☐ Daily ☐ Prefer not to answer

**39f. Does your incontinence affect your lifestyle?**

☐ Never ☐ Almost never ☐ 1–3 times per month ☐ 1–3 times per week ☐ Daily ☐ Prefer not to answer

**40. Have you needed to seek medical attention in the postnatal period due to medical issues/complications**?

☐ No ☐ Yes ☐ Can't / don't want to answer

**41. Were you treated with antibiotics for any infection after giving birth?**

☐ No ☐ Yes, urinary tract infection ☐ Yes, uterine infection ☐ Yes, wound infection ☐ Yes, breast infection ☐ Yes, other infection ☐ Cannot / do not wish to answer

**42. If you have, or have had, problems breastfeeding – have you received the amount of support you wanted from the healthcare services?**

☐ 1 No, not at all ☐ 2 ☐ 3 ☐ 4 ☐ 5 Yes, completely ☐ Can't / Cannot / do not wish to answer ☐ Not applicable

**43. Do you think the healthcare services met your needs when you gave birth and after you gave birth?**

☐ 1 No, not at all ☐ 2 ☐ 3 ☐ 4 ☐ 5 Yes, completely ☐ Cannot / do not wish to answer

**44. It isn’t possible to cover everything in a questionnaire. Do you have any views you want to share with us, or do you want to answer in more detail?**
