# Supplementary material for: Medical Interventions and Women's Perceptions of Respectful Intrapartum Care: A National Survey‐Based Cohort Study
Source: BJOG. 2025 Aug 11;132(12):1844–55. doi: 10.1111/1471-0528.18329 (PMC12501740; doi:10.1111/1471-0528.18329)
Supplement: Supplementary file 3 — Table S1: bjo18329‐sup‐0003‐TableS1.docx. [file BJO-132-1844-s001.docx]

Table S1. Comparison between responders and non-responders to the NPS-8 by study population characteristics, Sweden, 2022-2023.

|  | Responders | Non-responders | *p* value |
| --- | --- | --- | --- |
|  | n= 20 363 | n= 13 748 |  |
|  | n (%) | n (%) |  |
| Age (years), mean (SD) | 30.1 (4.2) | 29.2 (4.6) |  |
| Age categories |  |  | <0.001 |
| <25 | 2295 (11.3) | 2570 (18.7) |  |
| 25-29 | 7553 (37.1) | 5267 (38.3) |  |
| 30-34 | 8052 (39.5) | 4470 (32.5) |  |
| ≥35 | 2463 (12.1) | 1440 (10.5) |  |
|  |  |  |  |
| BMI (kg/m²), mean (SD) | 24.6 (4.5) | 24.2 (4.2) |  |
| BMI categories |  |  | <0.001 |
| Underweight (<18.5) | 466 (2.3) | 492 (3.6) |  |
| Normal (18.5-24.9) | 11 889 (58.4) | 8130 (59.1) |  |
| Overweight (25-29.9) | 4913 (24.1) | 3129 (22.8) |  |
| Obese (≥30) | 2208 (10.8) | 1276 (9.3) |  |
| Missing data | 887 (4.4) | 721 (5.2) |  |
|  |  |  |  |
| Level of education |  |  | <0.001 |
| Elementary school | 410 (2.0) | 804 (5.9) |  |
| Upper secondary school | 5113 (25.1) | 3920 (28.5) |  |
| Universityᵃ | 12 123 (59.5) | 6313 (45.9) |  |
| Missing data | 2717 (13.3) | 2711 (19.7) |  |
|  |  |  |  |
| Living with partner |  |  | <0.001 |
| Yes | 18 358 (90.2) | 11 942 (86.9) |  |
| Missing data | 674 (3.3) | 529 (3.9) |  |
|  |  |  |  |
| Country of birth |  |  | <0.001 |
| Nordic | 15 766 (77.4) | 8136 (59.2) |  |
| Non-Nordic | 2928 (14.4) | 4014 (29.2) |  |
| Missing data | 1669 (8.2) | 1598 (11.6) |  |
|  |  |  |  |
| Positive self-assessed health before pregnancy |  |  | 0.051 |
| Yes | 15 949 (78.3) | 10 040 (73.0) |  |
| Missing data | 3111 (15.3) | 2812 (20.5) |  |
|  |  |  |  |
| Pre-pregnancy comorbidityᵇ |  |  | <0.001 |
| Yes | 4101 (20.1) | 2416 (17.6) |  |
|  |  |  |  |
| Pregnancy comorbidityᵈ |  |  | 0.304 |
| Yes | 773 (3.8) | 552 (4.0) |  |
|  |  |  |  |
| Mental illness |  |  | <0.001 |
| Yes | 5238 (25.7) | 2974 (21.6) |  |
| Missing data | 361 (1.8) | 298 (2.2) |  |
|  |  |  |  |
| Fear of birth |  |  | 0.347 |
| Yes | 1440 (7.1) | 958 (7.0) |  |
| Missing data | 2437 (12.0) | 2262 (16.5) |  |
|  |  |  |  |
| Epidural analgesia |  |  | 0.193 |
| Yes | 11 834 (58.1) | 8087 (58.8) |  |
|  |  |  |  |
| Oxytocin augmentation |  |  | 0.333 |
| Yes | 11 643 (57.2) | 7788 (56.7) |  |
|  |  |  |  |
| Episiotomy |  |  | 0.726 |
| Yes | 1276 (6.3) | 853 (6.2) |  |
| Missing data | 656 (3.2) | 375 (2.7) |  |
|  |  |  |  |
| Mode of birth |  |  | 0.301 |
| Spontaneous vaginal | 17 885 (87.8) | 12 126 (88.2) |  |
| Instrumental | 2478 (12.2) | 1622 (11.8) |  |
|  |  |  |  |
| Postpartum haemorrhage (≥1000 ml) |  |  | 0.019 |
| Yes | 1672 (8.2) | 1033 (7.5) |  |
|  |  |  |  |
| Apgar <7 at 5 min |  |  | 0.792 |
| Yes | 218 (1.1) | 143 (1.0) |  |
| Missing data | 67 (0.3) | 54 (0.4) |  |
|  |  |  |  |
| Adverse neonatal outcome |  |  | 0.552 |
| Yes | 396 (1.9) | 255 (1.9) |  |
|  |  |  |  |
| Birth weight (g), mean (SD) | 3478.9 (418.6) | 3435.6 (422.2) |  |
| Birth weight categories |  |  | <0.001 |
| <3000 | 2451 (12.0) | 2057 (15.0) |  |
| 3000-3499 | 8316 (40.8) | 5739 (41.7) |  |
| 3500-3999 | 7298 (35.8) | 4623 (33.6) |  |
| ≥4000 | 2222 (10.9) | 1278 (9.3) |  |
| Missing data | 76 (0.4) | 51 (0.4) |  |
|  |  |  |  |
| Gestational age at birth (weeks + days) |  |  | 0.005 |
| 37+0-38+6 | 3489 (17.1) | 2462 (17.9) |  |
| 39+0-40+6 | 13 710 (67.3) | 9308 (67.7) |  |
| ≥41+0 | 3164 (15.5) | 1975 (14.4) |  |
|  |  |  |  |
| Hospital sizeᵉ |  |  | <0.001 |
| <1000 | 1335 (6.6) | 747 (5.4) |  |
| 1000-1999 | 4697 (23.1) | 2646 (19.3) |  |
| 2000-2999 | 3770 (18.5) | 2603 (19.0) |  |
| ≥3000 | 3962 (19.5) | 2640 (19.2) |  |
| University hospital | 6595 (32.4) | 5102 (37.1) |  |

**ᵃ**3-5 years

**ᵇ**Hypertension, diabetes type 1 or 2, systemic lupus erythematosus (SLE), renal conditions, and epilepsy

**ᵈ**Gestational diabetes, preeclampsia, and hepathosis

ᵉAnnual birth volume and teaching status (44 labour wards included)
