# Supplementary material for: Medical Interventions and Women's Perceptions of Respectful Intrapartum Care: A National Survey‐Based Cohort Study
Source: BJOG. 2025 Aug 11;132(12):1844–55. doi: 10.1111/1471-0528.18329 (PMC12501740; doi:10.1111/1471-0528.18329)
Supplement: Supplementary file 4 — Table S2: bjo18329‐sup‐0004‐TableS2.docx. [file BJO-132-1844-s003.docx]

Table S2. Maternal and labour characteristics of the study population, comparison between responders and non-responders for each of the outcome variables of the NPS-8. Primiparous women with spontaneous labour onset and vaginal births, Sweden, 2022-2023.

|  | Treated respectfully* | | *p* value | Received support* | | *p* value | Adequately informed* | | *p* value | Involved in  decision-making* | | *p* value |
| --- | --- | --- | --- | --- | --- | --- | --- | --- | --- | --- | --- | --- |
|  | Responders | Non-responders |  | Responders | Non-responders |  | Responders | Non-responders |  | Responders | Non-responders |  |
|  | n= 18 000 | n= 994 |  | n= 18 054 | n= 940 |  | n= 17 841 | n= 1153 |  | n= 17 672 | n= 1322 |  |
|  | n (%) | n (%) |  | n (%) | n (%) |  | n (%) | n (%) |  | n (%) | n (%) |  |
| Age (years), mean (SD) | 30.2 (4.2) | 29.0 (4.5) |  | 30.2 (4.2) | 29.0 (4.6) |  | 30.2 (4.2) | 29.1 (4.5) |  | 30.2 (4.2) | 29.2 (4.5) |  |
| Age categories |  |  | <0.001 |  |  | <0.001 |  |  | <0.001 |  |  | <0.001 |
| <25 | 1868 (10.4) | 176 (17.7) |  | 1868 (10.4) | 176 (17.7) |  | 1840 (10.3) | 204 (17.7) |  | 1821 (10.3) | 223 (16.9) |  |
| 25-29 | 6646 (36.9) | 408 (41.1) |  | 6646 (36.9) | 408 (41.1) |  | 6589 (36.9) | 465 (40.3) |  | 6534 (37.0) | 520 (39.3) |  |
| 30-34 | 7262 (40.3) | 318 (32.0) |  | 7262 (40.3) | 318 (32.0) |  | 7204 (40.4) | 376 (32.6) |  | 7129 (40.3) | 451 (34.1) |  |
| ≥35 | 2224 (12.4) | 92 (9.3) |  | 2224 (12.4) | 92 (9.3) |  | 2208 (12.4) | 108 (9.4) |  | 2188 (12.4) | 128 (9.7) |  |
|  |  |  |  |  |  |  |  |  |  |  |  |  |
| BMI (kg/m²), mean (SD) | 24.6 (4.5) | 24.5 (4.5) |  | 24.6 (4.5) | 24.4 (4.4) |  | 24.6 (4.5) | 24.7 (4.7) |  | 24.6 (4.5) | 24.7 (4.6) |  |
| BMI categories |  |  | 0.142 |  |  | 0.058 |  |  | 0.106 |  |  | 0.058 |
| Underweight (<18.5) | 403 (2.2) | 19 (1.9) |  | 403 (2.2) | 19 (2.0) |  | 401 (2.3) | 21 (1.8) |  | 397 (2.3) | 25 (1.9) |  |
| Normal (18.5-24.9) | 10 490 (58.3) | 607 (61.1) |  | 10 517 (58.3) | 580 (61.7) |  | 10 409 (58.3) | 688 (59.7) |  | 10 330 (58.5) | 767 (58.0) |  |
| Overweight (25-29.9) | 4378 (24.3) | 211 (21.2) |  | 4396 (24.4) | 193 (20.5) |  | 4341 (24.3) | 248 (21.5) |  | 4301 (24.3) | 288 (21.8) |  |
| Obese (≥30) | 1974 (11.0) | 107 (10.8) |  | 1981 (11.0) | 100 (10.6) |  | 1942 (10.9) | 139 (12.1) |  | 1914 (10.8) | 167 (12.6) |  |
| Missing data | 755 (4.2) | 50 (5.0) |  | 757 (4.2) | 48 (5.1) |  | 748 (4.2) | 57 (4.9) |  | 730 (4.1) | 75 (5.7) |  |
|  |  |  |  |  |  |  |  |  |  |  |  |  |
| Level of education |  |  | <0.001 |  |  | <0.001 |  |  | <0.001 |  |  | <0.001 |
| Elementary school | 306 (1.7) | 31 (3.1) |  | 307 (1.7) | 30 (3.2) |  | 303 (1.7) | 34 (3.0) |  | 294 (1.7) | 43 (3.3) |  |
| Upper secondary school | 4400 (24.4) | 312 (31.4) |  | 4410 (24.4) | 302 (32.1) |  | 4335 (24.3) | 377 (32.7) |  | 4300 (24.3) | 412 (31.2) |  |
| Universityᵃ | 10 992 (61.1) | 482 (48.5) |  | 11 021 (61.1) | 453 (48.2) |  | 10 918 (61.2) | 556 (48.2) |  | 10 828 (61.3) | 646 (48.9) |  |
| Missing data | 2302 (12.8) | 169 (17.0) |  | 2316 (12.8) | 155 (16.5) |  | 2285 (12.8) | 186 (16.1) |  | 2250 (12.7) | 221 (16.7) |  |
|  |  |  |  |  |  |  |  |  |  |  |  |  |
| Living with partner |  |  | 0.027 |  |  | 0.270 |  |  | 0.007 |  |  | <0.001 |
| Yes | 16 310 (90.6) | 877 (88.2) |  | 16 352 (90.6) | 835 (88.8) |  | 16 171 (90.6) | 1016 (88.1) |  | 16 040 (90.8) | 1147 (86.8) |  |
| Missing data | 566 (3.1) | 38 (3.8) |  | 566 (3.1) | 38 (4.0) |  | 561 (3.1) | 43 (3.7) |  | 546 (3.1) | 58 (4.4) |  |
|  |  |  |  |  |  |  |  |  |  |  |  |  |
| Country of birth |  |  | <0.001 |  |  | <0.001 |  |  | <0.001 |  |  | <0.001 |
| Nordic | 14 335 (79.6) | 633 (63.7) |  | 14 367 (79.6) | 601 (63.9) |  | 14 207 (79.6) | 761 (66.0) |  | 14 093 (79.8) | 875 (66.2) |  |
| Non-Nordic | 2247 (12.5) | 267 (26.9) |  | 2261 (12.5) | 253 (26.9) |  | 2226 (12.5) | 288 (25.0) |  | 2198 (12.4) | 316 (23.9) |  |
| Missing data | 1418 (7.9) | 94 (9.5) |  | 1426 (7.9) | 86 (9.2) |  | 1408 (7.9) | 104 (9.0) |  | 1381 (7.8) | 131 (9.9) |  |
|  |  |  |  |  |  |  |  |  |  |  |  |  |
| Positive self-assessed health  before pregnancy |  |  | 0.047 |  |  | 0.031 |  |  | 0.027 |  |  | 0.080 |
| Yes | 14 182 (78.8) | 753 (75.8) |  | 14 219 (78.8) | 716 (76.2) |  | 14 060 (78.8) | 875 (75.9) |  | 13 936 (78.9) | 999 (75.6) |  |
| Missing data | 2683 (14.9) | 164 (16.5) |  | 2698 (15.0) | 149 (15.9) |  | 2659 (14.9) | 188 (16.3) |  | 2621 (14.8) | 226 (17.1) |  |
|  |  |  |  |  |  |  |  |  |  |  |  |  |
| Pre-pregnancy comorbidityᵇ |  |  | 0.674 |  |  | 0.305 |  |  | 0.332 |  |  | 0.150 |
| Yes | 3630 (20.2) | 195 (19.6) |  | 3648 (20.2) | 177 (18.8) |  | 3580 (20.1) | 245 (21.3) |  | 3579 (20.3) | 246 (18.6) |  |
|  |  |  |  |  |  |  |  |  |  |  |  |  |
| Pregnancy comorbidityᵈ |  |  | 0.126 |  |  | 0.116 |  |  | 0.011 |  |  | 0.116 |
| Yes | 663 (3.7) | 46 (4.6) |  | 665 (3.7) | 44 (4.7) |  | 650 (3.6) | 59 (5.1) |  | 665 (3.7) | 44 (4.7) |  |
|  |  |  |  |  |  |  |  |  |  |  |  |  |
| Mental illness |  |  |  |  |  |  |  |  | 0.568 |  |  | 0.203 |
| Yes | 4663 (25.9) | 246 (24.8) | 0.522 | 4678 (25.9) | 231 (24.6) | 0.448 | 4605 (25.8) | 304 (26.4) |  | 4590 (26.0) | 319 (24.1) |  |
| Missing data | 299 (1.7) | 26 (2.6) |  | 301 (1.7) | 24 (2.6) |  | 297 (1.7) | 28 (2.4) |  | 290 (1.6) | 35 (2.7) |  |
|  |  |  |  |  |  |  |  |  |  |  |  |  |
| Fear of birth |  |  | 0.388 |  |  | 0.507 |  |  | 0.899 |  |  | 0.113 |
| Yes | 1279 (7.1) | 61 (6.1) |  | 1281 (7.1) | 59 (6.3) |  | 1262 (7.1) | 78 (6.8) |  | 1264 (7.2) | 76 (5.8) |  |
| Missing data | 2066 (11.5) | 147 (14.8) |  | 2074 (11.5) | 139 (14.8) |  | 2050 (11.5) | 163 (14.1) |  | 2017 (11.4) | 196 (14.8) |  |
|  |  |  |  |  |  |  |  |  |  |  |  |  |
| Epidural analgesia |  |  | 0.012 |  |  | 0.017 |  |  | 0.454 |  |  | <0.001 |
| Yes | 10 372 (57.6) | 613 (61.7) |  | 10 406 (57.6) | 579 (61.6) |  | 10 306 (57.8) | 679 (58.9) |  | 10 301 (58.3) | 684 (51.7) |  |
|  |  |  |  |  |  |  |  |  |  |  |  |  |
| Oxytocin augmentation |  |  | 0.266 |  |  | 0.417 |  |  | 0.027 |  |  | <0.001 |
| Yes | 10 319 (57.3) | 552 (55.5) |  | 10 345 (57.3) | 526 (56.0) |  | 10 247 (57.4) | 624 (54.1) |  | 10 229 (57.9) | 642 (48.6) |  |
|  |  |  |  |  |  |  |  |  |  |  |  |  |
| Episiotomy |  |  | 0.161 |  |  | 0.177 |  |  | 0.073 |  |  | 0.158 |
| Yes | 1144 (6.4) | 53 (5.3) |  | 1147 (6.4) | 50 (5.3) |  | 1138 (6.4) | 59 (5.1) |  | 1125 (6.4) | 72 (5.5) |  |
| Missing data | 594 (3.3) | 19 (1.9) |  | 592 (3.3) | 21 (2.2) |  | 587 (3.3) | 26 (2.3) |  | 582 (3.3) | 31 (2.3) |  |
|  |  |  |  |  |  |  |  |  |  |  |  |  |
| Mode of birth |  |  | 0.187 |  |  | 0.195 |  |  | 0.146 |  |  | 0.326 |
| Spontaneous vaginal | 15 791 (87.7) | 886 (89.1) |  | 15 839 (87.7) | 838 (89.2) |  | 15 649 (87.7) | 1028 (89.2) |  | 15 505 (87.7) | 1172 (88.7) |  |
| Instrumental | 2209 (12.3) | 108 (10.9) |  | 2215 (12.3) | 102 (10.9) |  | 2192 (12.3) | 125 (10.8) |  | 2167 (12.3) | 150 (11.4) |  |
|  |  |  |  |  |  |  |  |  |  |  |  |  |
| Postpartum haemorrhage  (≥1000 ml) |  |  | 0.012 |  |  | 0.070 |  |  | 0.141 |  |  | 0.003 |
| Yes | 1507 (8.4) | 61 (6.1) |  | 1505 (8.3) | 63 (6.7) |  | 1486 (8.3) | 82 (7.1) |  | 1487 (8.4) | 81 (6.1) |  |
|  |  |  |  |  |  |  |  |  |  |  |  |  |
| Apgar <7 at 5 min |  |  | 0.272 |  |  | 0.542 |  |  | 0.356 |  |  | 0.277 |
| Yes | 193 (1.1) | 7 (0.7) |  | 192 (1.1) | 8 (0.9) |  | 191 (1.1) | 9 (0.8) |  | 190 (1.1) | 10 (0.8) |  |
| Missing data | 59 (0.3) | 6 (0.6) |  | 58 (0.3) | 7 (0.7) |  | 57 (0.3) | 8 (0.7) |  | 57 (0.3) | 8 (0.6) |  |
|  |  |  |  |  |  |  |  |  |  |  |  |  |
| Adverse neonatal outcome |  |  | 0.618 |  |  | 0.988 |  |  | 0.798 |  |  | 0.933 |
| Yes | 348 (1.9) | 17 (1.7) |  | 347 (1.9) | 18 (1.9) |  | 344 (1.9) | 21 (1.8) |  | 340 (1.9) | 25 (1.9) |  |
|  |  |  |  |  |  |  |  |  |  |  |  |  |
| Birth weight (g), mean (SD) | 3484.4 (417.6) | 3437.4 (423.1) |  | 3483.9 (417.7) | 3444.1 (421.8) |  | 3484.6 (417.7) | 3439.8 (421.2) |  | 3485.9 (416.7) | 3428.2 (431.2) |  |
| Birth weight categories |  |  | 0.001 |  |  | 0.008 |  |  | 0.001 |  |  | <0.001 |
| <3000 | 2083 (11.6) | 156 (15.7) |  | 2095 (11.6) | 144 (15.3) |  | 2060 (11.6) | 179 (15.5) |  | 2020 (11.4) | 219 (16.6) |  |
| 3000-3499 | 7356 (40.9) | 401 (40.3) |  | 7385 (40.9) | 372 (39.6) |  | 7294 (40.9) | 463 (40.2) |  | 7233 (40.9) | 524 (39.6) |  |
| 3500-3999 | 6513 (36.2) | 328 (33.0) |  | 6520 (36.1) | 321 (34.2) |  | 6456 (36.2) | 385 (33.4) |  | 6393 (36.2) | 448 (33.9) |  |
| ≥4000 | 1980 (11.0) | 107 (10.8) |  | 1986 (11.0) | 101 (10.7) |  | 1963 (11.0) | 124 (10.8) |  | 1961 (11.1) | 126 (9.5) |  |
| Missing data | 68 (0.4) | 2 (0.2) |  | 68 (0.4) | 2 (0.2) |  | 68 (0.4) | 2 (0.2) |  | 65 (0.4) | 5 (0.4) |  |
|  |  |  |  |  |  |  |  |  |  |  |  |  |
| Gestational age at birth  (weeks + days) |  |  | 0.463 |  |  | 0.384 |  |  | 0.067 |  |  | 0.001 |
| 37+0-38+6 | 3077 (17.1) | 171 (17.2) |  | 3088 (17.1) | 160 (17.0) |  | 3037 (17.0) | 211 (18.3) |  | 2990 (17.1) | 258 (19.5) |  |
| 39+0-40+6 | 12 108 (67.3) | 682 (68.6) |  | 12 141 (67.3) | 649 (69.0) |  | 12 001 (67.3) | 789 (68.4) |  | 11 891 (67.3) | 899 (68.0) |  |
| ≥41+0 | 2815 (15.6) | 141 (14.2) |  | 2825 (15.7) | 131 (13.9) |  | 2803 (15.7) | 153 (13.3) |  | 2791 (15.8) | 165 (12.5) |  |

*Outcome variables from NPS-8: During labour and birth, did the caregivers treat you with respect? / During labour and birth, did you receive support from the caregivers to the extent you desired? / During labour and birth, did you receive enough information? / During labour and birth, were you involved in planning and decision-making to the extent you desired?

All outcome variables were rated on a 5-point Likert scale and dichotomised accordingly: agree (response options 4-5), disagree (response options 1-3).

ᵇHypertension, diabetes type 1 or 2, systemic lupus erythematosus (SLE), renal conditions, and epilepsy

ᵈGestational diabetes, preeclampsia, and hepathosis
