# Supplementary material for: Medical Interventions and Women's Perceptions of Respectful Intrapartum Care: A National Survey‐Based Cohort Study
Source: BJOG. 2025 Aug 11;132(12):1844–55. doi: 10.1111/1471-0528.18329 (PMC12501740; doi:10.1111/1471-0528.18329)
Supplement: Supplementary file 5 — Table S3: bjo18329‐sup‐0005‐TableS3.docx. [file BJO-132-1844-s004.docx]

Table S3. Adjusted odds ratios (aOR) with 95% confidence intervals (CI) for primiparous women with and without PPH (postpartum haemorrhage ≥1000 ml)

|  | Treated respectfully | | | |
| --- | --- | --- | --- | --- |
|  | PPH included (N=18 000) | | PPH excluded (N= 16 416) | |
|  | n (%) | aOR (95% CI) | n (%) | aOR (95% CI) |
| No epidural | 7135/7628 (93.5) | 1.0 | 6546/7001 (93.5) | 1.0 |
| Epidural | 9753/10372 (94.0) | 1.23 (1.06-1.41) | 8882/9415 (94.3) | 1.25 (1.07-1.46) |
|  |  |  |  |  |
| No oxytocin | 7196/7681 (93.7) | 1.0 | 6769/7210 (93.9) | 1.0 |
| Oxytocin | 9692/10319 (93.9) | 0.98 (0.85-1.13) | 8677/9206 (94.3) | 1.01 (0.87-1.18) |
|  |  |  |  |  |
| No episiotomy | 15275/16262 (93.9) | 1.0 | 14037/14902 (94.2) | 1.0 |
| Episiotomy | 1070/1144 (93.5) | 0.91 (0.69-1.22) | 941/1007 (93.4) | 0.88 (0.65-1.19) |
|  |  |  |  |  |
|  | **Received support** | | | |
|  | PPH included (N= 18 054) | | PPH excluded (N= 16 471) | |
|  | n (%) | aOR (95% CI) | n (%) | aOR (95% CI) |
| No epidural | 6760/7648 (88.4) | 1.0 | 6231/7023 (88.7) | 1.0 |
| Epidural | 9205/10406 (88.5) | 1.05 (0.95-1.17) | 8380/9448 (88.7) | 1.04 (0.93-1.16) |
|  |  |  |  |  |
| No oxytocin | 6955/7709 (90.2) | 1.0 | 6543/7237 (90.4) | 1.0 |
| Oxytocin | 9010/10345 (87.1) | 0.72 (0.64-0.80) | 8068/9234 (87.4) | 0.73 (0.65-0.82) |
|  |  |  |  |  |
| No episiotomy | 14448/16315 (88.6) | 1.0 | 13292/14955 (88.9) | 1.0 |
| Episiotomy | 996/1147 (86.8) | 0.79 (0.64-0.97) | 869/1010 (86.0) | 0.70 (0.56-0.86) |
|  |  |  |  |  |
|  | **Adequately informed** | | | |
|  | PPH included (N= 17 841) | | PPH excluded (N= 16 280) | |
|  | n (%) | aOR (95% CI) | n (%) | aOR (95% CI) |
| No epidural | 6225/7535 (82.6) | 1.0 | 5747/6918 (83.1) | 1.0 |
| Epidural | 8545/10306 (82.9) | 1.03 (0.94-1.13) | 7805/9362 (83.4) | 1.03 (0.94-1.14) |
|  |  |  |  |  |
| No oxytocin | 6431/7594 (84.7) | 1.0 | 6058/7128 (85.0) | 1.0 |
| Oxytocin | 8339/10247 (81.4) | 0.75 (0.68-0.82) | 7494/9152 (81.9) | 0.76 (0.69-0.84) |
|  |  |  |  |  |
| No episiotomy | 13420/16116 (83.3) | 1.0 | 12369/14776 (83.7) | 1.0 |
| Episiotomy | 882/1138 (77.5) | 0.69 (0.58-0.82) | 777/1002 (77.5) | 0.67 (0.56-0.80) |
|  |  |  |  |  |
|  | **Involved in decision-making** | | | |
|  | PPH included (N= 17 672) | | PPH excluded (N= 16 110) | |
|  | n (%) | aOR (95% CI) | n (%) | aOR (95% CI) |
| No epidural | 5957/7371 (80.8) | 1.0 | 5504/6760 (81.4) | 1.0 |
| Epidural | 8469/10301 (82.2) | 1.18 (1.08-1.29) | 7746/9350 (82.8) | 1.19 (1.09-1.31) |
|  |  |  |  |  |
| No oxytocin | 6250/7443 (84.0) | 1.0 | 5879/6986 (84.2) | 1.0 |
| Oxytocin | 8176/10229 (79.9) | 0.77 (0.70-0.84) | 7371/9124 (80.8) | 0.79 (0.72-0.88) |
|  |  |  |  |  |
| No episiotomy | 13120/15965 (82.2) | 1.0 | 12102/14625 (82.7) | 1.0 |
| Episiotomy | 838/1125 (74.5) | 0.59 (0.51-0.70) | 742/988 (75.1) | 0.57 (0.48-0.68) |
|  |  |  |  |  |

Adjusted for age, BMI, country of birth, level of education, mental illness, positive self-assessed health before pregnancy, fear of birth, pre-pregnancy comorbidity, pregnancy comorbidity, gestational age at birth, and hospital size
